# Seq2pathway: an R/Bioconductor package for pathway analysis of next-generation sequencing data

**DOI:** 10.1093/bioinformatics/btv289

**Published:** 2015-05-15

**Authors:** Bin Wang, John M. Cunningham, Xinan (Holly) Yang

**Affiliations:** Section of Hematology/Oncology, Department of Pediatrics, The University of Chicago, Chicago, IL, USA

## Abstract

**Summary**: Seq2pathway is an R/Python wrapper for pathway (or functional gene-set) analysis of genomic loci, adapted for advances in genome research. Seq2pathway associates the biological significance of genomic loci with their target transcripts and then summarizes the quantified values on the gene-level into pathway scores. It is designed to isolate systematic disturbances and common biological underpinnings from next-generation sequencing (NGS) data. Seq2pathway offers Bioconductor users enhanced capability in discovering collective pathway effects caused by both coding genes and cis-regulation of non-coding elements.

**Availability and implementation:** The package is freely available at http://www.bioconductor.org/packages/release/bioc/html/seq2pathway.html.

**Contact**: xyang2@uchicago.edu

**Supplementary information:** Supplementary data are available at *Bioinformatics* online.

## 1 Introduction

Functional gene-set analysis (fGSA) improves biological interpretability of genomic features with respect to prior knowledge and has led to important biological discoveries (Khatri *et al.*, 2012). Its power lies in the fact that it detects statistical significance over an entire dataset of gene-sets and yields biologically meaningful interpretations (Subramanian *et al.*, 2005). Recently, researchers revealed driver pathways that predispose individuals to disease (Vandin *et al.*, 2012) and shed light on the development of targeted therapy (Gonzalez-Perez *et al.*, 2013) by performing fGSA on genes proximal to significantly disease-associated loci. However, with the increasing growth of genomic sequencing measurements, non-coding regulatory loci pose a critical challenge to fGSA, as nearly 99% of the nucleotides in the human genome do not code for proteins but instead richly harbor functional regulators of genes (Kellis *et al.*, 2014).

‘Gene-based’ fGSA methods neglect non-coding regulatory information because they have no readily annotated gene function and cannot be assigned to a specific biological pathway. For example, HOMER (Hypergeometric Optimization of Motif EnRichment), a popular tool for discovering cis-regulatory regions before any subsequent fGSA, links genomic loci to only the nearest genes (Wang *et al.*, 2011). Other fGSA algorithms take into account single-nucleotide polymorphisms (SNPs) only located in or near coding genes (Koster *et al.*, 2014; Nam *et al.*, 2010) or exonic mutations (Boca *et al.*, 2010; Gonzalez-Perez *et al.*, 2013; Leiserson *et al.*, 2013).

‘Sequence-derived’ fGSA regarding the functional impact of not only coding genes, but also non-coding regulators, is necessary. A growing body of evidence supports the strategy of connecting genomic loci and coding genes using a many-to-many mapping and considering long-distance cis-regulation. First, non-coding regions richly harbor functional regulatory elements in humans (Kellis *et al.*, 2014; Schierding *et al.*, 2014). Second, nearly half of the disease-associated SNPs are located in gene deserts (Visel *et al.*, 2009). Third, our recent study suggests that a disease-associated SNP located in an intron could be the enhancer of a neighboring gene rather than of its host gene (van den Boogaard *et al.*, 2014).

The first sequence-derived fGSA method is GREAT (the Genomic Regions Enrichment of Annotations Tool) (McLean *et al.*, 2011). GREAT quantifies an fGSA score by determining if the total number of loci within the regulatory domain of genes in a gene-set is greater than expected. The ChIP-Enrich method then empirically adjusts the length of the gene body and its surrounding non-coding sequence (Welch *et al.*, 2014). However, both GREAT and ChIP-Enrich treat all loci equally, despite the fact that a threshold for significance is always experimentally context-dependent and arbitrary.

We propose a new sequence-derived fGSA tool named ‘seq2pathway’ that fills the non-coding region gap in fGSA by considering quantitative sequencing measurements. We initially developed FAIME (Functional Analysis of Individual Microarray/RNAseq Expression) to compare the cumulative quantitative effects of genes inside an ontology (set of functionally related genes) with those outside, thus overcoming a number of difficulties in prior fGSA methods (Yang *et al.*, 2012). We here provide the community a Bioconductor package that (i) quantifies the functional impact of both coding genes and non-coding loci, (ii) generalizes four fGSA methods, including FAIME, to variable next-generation sequencing (NGS) data and (iii) wraps gene locus definitions for human and mouse genomes from the GENCODE project (Harrow *et al.*, 2012) into R objects.

## 2 Main features

Seq2pathway is designed for knowledge discovery using a variety of NGS data (e.g. ChIP-seq, RNA-seq, SNPs, etc) by taking the contribution of non‐coding loci and their experimental significance scores into consideration. Users can apply the ‘seq2pathway’ function jointly, or in a two-step algorithm consisting of ‘seq2gene’ and ‘gene2pathway’ components separately (For workflow see Supplementary Fig. S1).

The seq2gene step links both coding and non-coding regions to coding genes in a many-to-many mapping (For detailed Pseudo code see Supplementary Fig. S2) (Yang *et al.*, 2015b). Using seq2gene with a search radius of 100 kb, our recent study *in vivo* defined a novel cis-regulatory element from both ChIP-seq and transcriptomic data (Hoffmann *et al.*, 2014). Compared with other methods, seq2gene outputs candidate targets with detailed loci-to-gene mappings (e.g. exon, intron, CDS (CoDing sequence), UTR (UnTranslated Region), promoter and neighbor). This feature allows researchers to understand the function of the region and design creative downstream analyses for the genomic locus hits, an effort only previously achieved by annotation tools HOMER and ChIP-Enrich.

The gene2pathway step integrates several cutting-edge fGSA algorithms, characterized by the improved FAIME method (Supplementary Fig. S3). We also provide other three alternative methods: Fisher’s exact test, the Kolmogorov–Smirnov test, and the cumulative rank test (Subramanian *et al.*, 2005). All of these implemented analyses condense gene-by-sample measurements (gene profiles) to gene-set-by-sample measurements (gene-set profiles), which are gene-coverage-difference tolerable and free of gene-set size preference for gene-sets with five or more genes (Supplementary Figs S4A, S6A, and S8A).

Significance of biologically-defined loci of genomic aberrations or functional elements has been lacking of attention than their recurrence. Confidence of threshold-based significance is likely to depend on the choice of genomic background and the test statistic; thus, empirical approaches are recommended (Glaab *et al.*, 2012). Seq2pathway calculates empirical *P*-values for each gene-set in a sample by shuffling the gene scores derived from genomic locus hits of this sample to generate gene-set scores from the null hypothesis. This empirical assessment overcomes common biases of gene length in pathway analysis of NGS data (Supplementary Figs S4B and S6B).

Additionally, we collapsed significant scores per gene when a gene was mapped to multiple neighboring genomic loci, removing the bias of linkage disequilibrium for the downstream pathway analysis (Supplementary Fig. S6C).

Finally, researchers can run seq2pathway against user-supplied gene-sets and the wrapped Gene Ontology (GO). This feature essentially allows unbiased comparisons of methods and results.

## 3 Case study

To highlight the earlier features, we demonstrate the application of seq2pathway on a variety of different NGS datasets, point out the novel insights obtained only by seq2pathway, and summarize the take home message (Supplementary Document).

We first compared seq2pathway with ChIP-Enrich and GREAT to test GO enrichment in a set of H3K27me3 peaks using the same criteria (Supplementary Fig. S5, false discovery rate (FDR) < 0.01, count ≥5 and search radius = 5 kb). Both seq2pathway and GREAT identified the biological process GO:0008285 (negative regulation of cell proliferation), while seq2pathway concentrates better with less identified GO terms than the other two methods. Interestingly, the significance of GO:0003705 (distal enhancer region for RNA polymerase II), a molecular function significantly enriched by bivalent genes marked with both H3K4me3 and H3K27me3 (Li *et al.*, 2013), was obtained only with seq2pathway (Supplementary Fig. S5B).

**Fig. 1.**
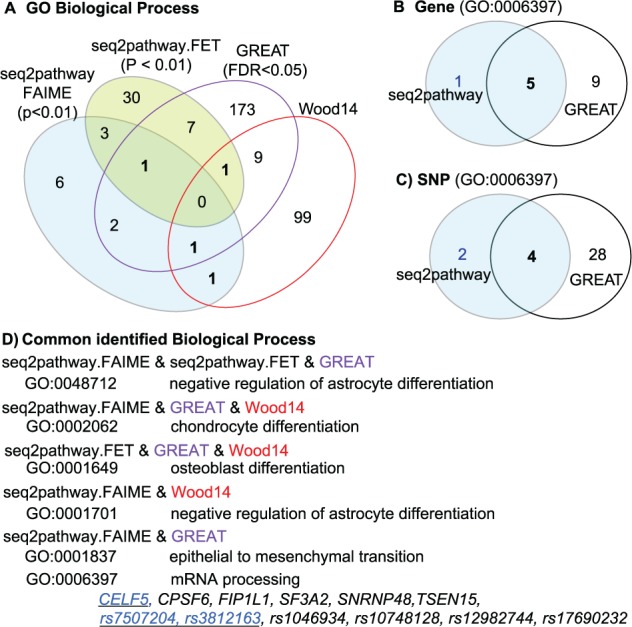
The identification of biological process from 460 height-associated SNPs. (**A**) Results of both methods were comparable to the prediction of a comprehensive study on human height (Wood *et al.*, 2014). (**B**) A demonstration of the case that both common and different genes are identified by two methods for the same GO term (GO:0006397). (**C**) Common and different SNPs are identified by two methods for the genes in Panel B. (**D**) Selective biological process terms identified by two or three methods, followed by the commonly identified (in black) and seq2pathway specifically identified (in blue, underlined) genes and SNPs shown in panels B–C.

We next tested 460 human height-associated SNP loci and compared the output with the seq2pathway (setting the parameter SNP = T) to the results of GREAT (Binomial FDR < 0.05, observed regions >3, region fold enrich >2). We applied a search radius = 5 kb for both methods. The valuable biological processes obtained only with seq2pathway include GO:0001701 (*in utero* embryonic development) that was predicted recently (Fig. 1) in a comprehensive study on human height (Wood *et al.*, 2014).

When we applied seq2pathway to RNA-seq data, individualized gene-set profiles allow the derivation of statistics at the pathway level directly rather than at the gene level. The advantages of seq2pathway lie in noise and dimension reduction, its desired biological interpretability, as well as a platform for novel integrative systems biology analysis (Yang *et al.*, 2015a)

## 4 Conclusion

It is a breakthrough in genome analytics coordinates to assign region significance scores such as binding affinity estimates from ChIP-Seq onto a uniform gene-set scale. This feature facilitates the integrative analysis of multiple datasets (Yang *et al.*, 2015a). The most critical issue in functionally interpreting genomic loci is to bridge non-coding regions with gene function. Seq2pathway offers the capacity to discover collective pathway effects caused by long-distance cis-regulation of non-coding elements. Importantly, seq2pathway enhances an alternative tool to integrate a number of ‘omics’ datasets into a condensed space of quantitative gene-set scores. This functional level integration will help generate assumptions, constraints, and interpretation in systems biology.

## Supplementary Material

Supplementary Data
