# Supplementary material for: Seq2pathway: an R/Bioconductor package for pathway analysis of next-generation sequencing data
Source: Bioinformatics. 2015 May 15;31(18):3043–5. doi: 10.1093/bioinformatics/btv289 (PMC4565027; doi:10.1093/bioinformatics/btv289)
Supplement: Supplementary Data [file supp_31_18_3043__index.html]

Seq2pathway: an R/Bioconductor package for pathway analysis of next-generation sequencing data — Seq2pathway: an R/Bioconductor package for pathway analysis of next-generation sequencing data — Seq2pathway: an R/Bioconductor package for pathway analysis of next-generation sequencing data — Supplementary Data 

# Seq2pathway: an R/Bioconductor package for pathway analysis of next-generation sequencing data

## Supplementary Data

files

- Supplementary Data - pdf file
