# Supplementary material for: Seq2pathway: an R/Bioconductor package for pathway analysis of next-generation sequencing data
Source: Bioinformatics. 2015 May 15;31(18):3043–5. doi: 10.1093/bioinformatics/btv289 (PMC4565027; doi:10.1093/bioinformatics/btv289)
Supplement: Supplementary Data [file supp_btv289_seq2pathwaypackage_Suppl.pdf]

Supplementary document for
Seq2pathway: an R/Bioconductor package for pathway analysis of next-generation sequencing data

Bin Wang, John M Cunningham, Xinan (Holly) Yang*

March 31, 2015

Contents

1 Introduction **2**

1.1 Main features . . . 2
1.2 seq2pathway flowchart, including seq2gene and gene2pathway components . . . 2
1.3 seq2gene pseudo-code . . . 3
1.4 gene2pathway algorithm . . . 4

2 Case study **6**

2.1 Data . . . 6
2.2 Highlights . . . 6
2.3 ChIP-seq data analysis . . . 7
2.3.1 Discover over-represented GO terms from H3K27me3 enriched peaks . . . 7
2.3.2 Overcome the bias of gene length and gene-set size . . . 7
2.3.3 Method comparison . . . 8
2.3.4 R code . . . 9
2.4 SNP data analysis . . . 12
2.4.1 Discover over-represented Disease Ontology terms from height-associated SNPs . . . 12
2.4.2 Overcoming the bias of gene length, gene-size, and linkage disequilibrium . . . 12
2.4.3 Method comparison . . . 14
2.4.4 R code . . . 14
2.5 RNA-seq data analysis . . . 17
2.5.1 AML samples . . . 18
2.5.2 Gene-set profile is independent of gene-set size . . . 18
2.5.3 Discover risk-dependent GO terms from AML patients with cytogenetic abnormalities. . . . 18
2.5.4 R code . . . 19
2.6 R environment session . . . 21

3 Take home points **22**

4 References of the supplementary material **23**

1 Introduction

Functional gene-set (including signaling pathways) enrichment analysis has been widely used for biological interpretation of next-generation sequencing (NGS) data[14]. We designed `seq2pathway` for novel functional analysis of NGS data by taking the effect of non-coding loci and their experimental significance scores into consideration. Applying `seq2pathway` to both ChIP-seq and transcriptomic data, our recent studies revealed novel cis-regulatory elements that explain genome-wide association study (GWAS) identification[27] or can be in vivo defined[8]. This R/Bioconductor package offers Bioconductor users enhanced capability to discover collective pathway effects caused by both coding genes and cis-regulation of non-coding elements.

1.1 Main features

The main features of `seq2pathway` are outlined in six points below that provide additional insights into the NGS data.

- "seq2pathway" is a two-step algorithm consisting of "seq2gene" and "gene2pathway" components. Users can run two steps jointly or separately.
- "seq2gene" can aggregate locus significance scores such as binding affinity from ChIP-seq[27] or odds ratio from GWAS into gene-level scores per sample, free of gene length bias.
- "gene2pathway" provides four gene-set enrichment analytic approaches (FAIME[26], KS-rank, cumulative-rank, and Fisher's exact test (FET)), free of gene-set bias.
- "seq2pathway" can estimate empirical significance of pathway enrichment, which overcomes the gene-set analytic bias caused by gene length or gene-set size[26].
- "seq2pathway" can analyze customized ontology inputs (GO, KEGG pathway, Disease Ontology, etc.) and, importantly, can test in-house defined ontologies in conjunction with public canonic pathways.
- "seq2pathway" calculates comparable pathway-level scores for the same sample (or samples in the same group), even measured by different NGS technologies, facilitating the development of systematic analysis of NGS.

These features are essential to develop systematic study addressing open questions in the field of NGS data mining. We have recently used `seq2pathway` derived individualized pathway scores to perform dynamic mechanism analysis among leukemic and hematopoietic stem cells and revealed a favorable prognostic signature for acute myeloid leukemia[28]. For ChIP-seq data, the distribution of binding affinity, which greatly depends on the peak calling algorithm, will result in a variable range of locus-level significance scores. `Seq2pathway` deals with this challenge of identifying signals across the entire spectrum of high confidence/enrichment (peaks) and low confidence/enrichment (noise) by taking into account a ranked list of pre-threshold significance scores. Specifically, `seq2pathway` aggregates log-transformed p-values from loci into gene-level scores and then ranks the gene-level scores genome-wide.

To highlight these features, we demonstrate the application of `seq2pathway` on a variety of different NGS datasets (ChIP-seq, GWAS or whole-genome sequencing identified SNPs, and RNA-seq) in the following `case study` section. We also provide the ontological resources and R code for these case studies. Finally, we summarize take home points for users to apply seq2pathway.

Instructions for installation, detailed functions, and functional parameters can be found in the online vignette of the `seq2pathway` package from Bioconductor.

1.2 seq2pathway flowchart, including seq2gene and gene2pathway components

To provide users with a straightforward understanding of how the two-step method works, we give a flowchart (Figure 1) explaining seq2pathway that consists of seq2gene and gene2pathway components.

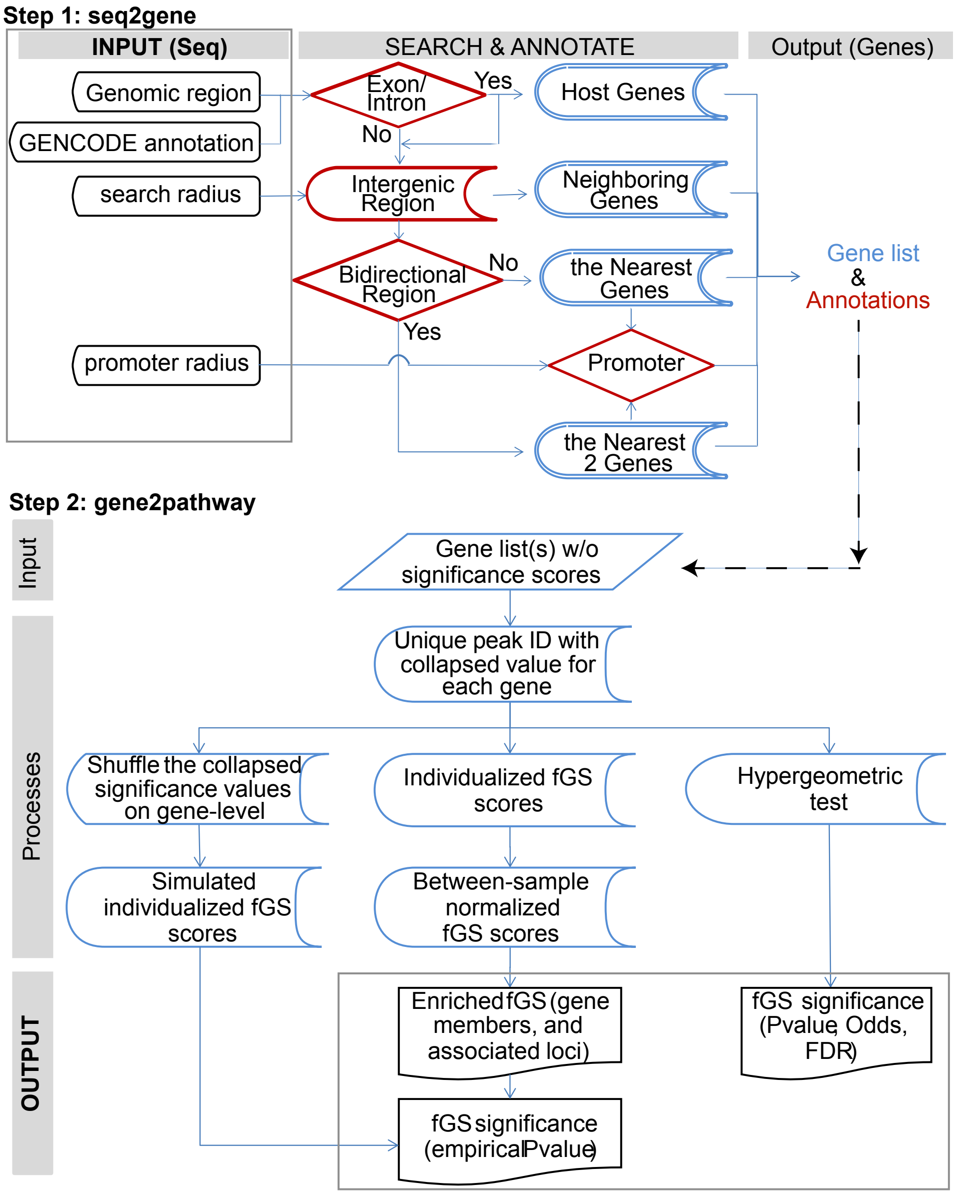

Figure 1: **seq2pathway two-step flowchart**. The inputs are on the top-left panel in square, and the outputs are on the bottom-right panel. A full pipeline analysis can be achieved by calling `runseq2pathway` in the package. Alternatively, users can call the two main functions `runseq2gene` and `gene2pathway_test` in series.

1.3 seq2gene pseudo-code

The seq2gene step links both coding and non-coding regions to coding genes in a many-to-many mapping[27]. Using seq2gene with a search radius of 100k bps, our recent in vivo study defined a novel cis-regulatory element from both ChIP-seq and transcriptomic data[8]. This function can find certain types of transcripts annotated by GENCODE in both the human and mouse genome.

Figure 2 is the pseudo code for the function seq2gene[27]. To perform the basic bisect algorithm with respect to exon and transcript separately, we used ENSEMBL IDs as the key index (Table 1).

Table 1: Statistics about the seq2pathway package used GENCODE annotation.

Species	GENCODE Release	Corresponding Ensembl assembly	# of coding genes	# of Long non-coding RNAs	# of Small non-coding RNAs	# of Pseudogenes	# of all genes
Human	19(Dec.2013)	GRCh74/hg19	20345	13870	9013	14206	57820
Mouse	M3(Apr.2014)	GRCm38/mm10	22026	5385	5853	7388	41128

Algorithm: seq2gene
Input: peaks, exontable, transcripttable, search radius
Output: peak with annotated gene information

```
for i:= 1 to length(peaks) do begin:
    m = peakleft
    n = peakright
    middle = (m+n)/2
    locate the nearest exon(J) for peak(i) by the basic bisect algorithm
    if peak(i) resides inside exon(J)
        report peak(i) with exon(J)
    endif
    while exon(x) intersecting with peak(i)
        report peak(i) with exon(x)
        exon(x) =the closest exons (left or right)
    endwhile
    locate the nearest transcript(H) by the basic bisect algorithm
    if peak(i) resides outside transcript(H)
        report peak(i) with transcript(H), intergenic region *
    else
        report peak(i) with transcript(H), intron region
    endif
    for transcripts(t) within the position of transcript(H)± search radius
        if peak(i) resides outside transcripts(t)
            report peak(i) with transcripts(x), intergenic region *
        else
            report peak(i) with transcripts(x), intron region
        endif
    end
end
```

***:** more details about distance, promoter and bidirectional region judgment

Figure 2: **Pseudo-code of the seq2gene algorithm [27]**.

1.4 gene2pathway algorithm

The analysis of gene-sets is superior to single-gene analysis in regard to noise and dimensional reduction, as well as its desired biological interpretability[17]. However, most gene-set analysis methods impose inherent limits on low cross-dataset comparison or reproducibility from gene-by-sample measurements, as only the genes measured by all

collected platforms can be interrogated together. Improved gene-set analyses condense transcriptomic data from gene-by-sample measurements (gene profiles) to gene-set-by-sample measurements (gene-set profiles), which are gene-coverage-difference tolerable and a breakthrough in genome analytics coordinates. Such gene-set-by-sample analyses facilitate the integration and analysis of multiple datasets, platforms, or layers of omics-data, by assigning them into a uniform gene-set scale. We apply the improved gene-set analysis method[26] here.

However, gene-set analyses are often prone to various sources of bias (e.g. gene length bias, gene-set size bias). The seq2pathway empirical p-value adjusts gene length bias by assigning the observed scores to all genes in the human genome randomly. The seq2pathway method also adjusts gene-set size bias by the calculation of a FAIME score[26](See Figure 3, Equation 2 for details).

Another concern for analysis of NGS datasets is that the assignment of significance scores varies greatly depending on the peak calling algorithm used in the analysis. We therefore applied a straightforward normalization (z-transfer) to gene-set scores that varied across samples and datasets. We have recently shown that the normalization enables multi-resource data integration and biologically functional interpretation by working on the same scale of functional gene-sets[28].

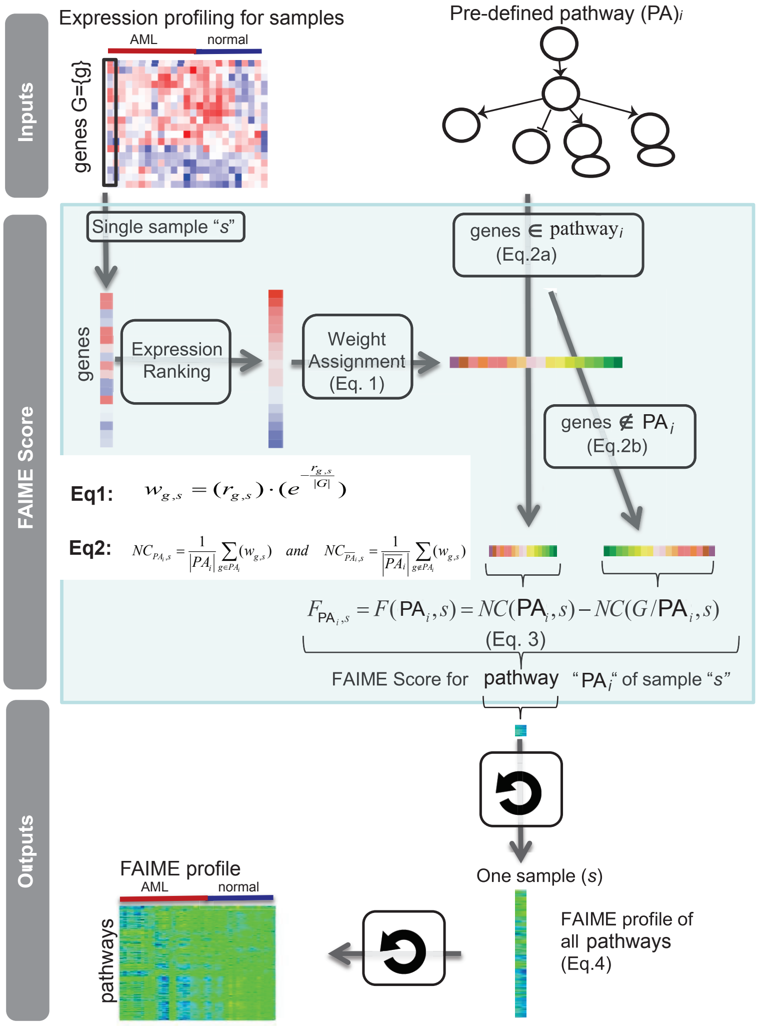

Figure 3: **Procedure for determining pathway profiles for each sample using microarray expression (FAIME profiles - Published in [26]).** There are three equations applied in series that calculate a gene-set score. The 2nd equation adjusts the gene-set size effects.

2 Case study

2.1 Data

Seq2pathway complements other NGS analytic approaches by providing Bioconductor users a new tool to put functional insight into NGS data systematically. To demonstrate its novelty, we perform the following case studies on a variety of different NGS datasets (ChIP-seq, GWAS or whole-genome sequencing identified SNPs, and RNA-seq) (Table 2).

Table 2: Public NGS data analyzed in the case study.

NGS	Number of loci (input)	Description	Linkage
ChIP-seq	41,464	H3K27me3 enrichment in GM12878 cells	ENCODE
SNP	460	Results from 21 studies in the search for "height".	NHGRI
RNA-seq	19,990	30 AML patients with cytogenetic abnormalities	TCGA

*: Number of genes processed by TCGA compendium.

Seq2pathway is further complementary to other approaches in terms of its studying on more customized knowledge database. Therefore in case studies using ChIP-seq and RNA-seq data, we demonstrate the test on GO databases. In the case study of SNP data, we demonstrate the test on the Human Disease Ontology database and the Reactome pathway database (Table 3).

Note that the MSigDB curated GO omitted gene-sets for very broad categories (such as Biological Processes) and very specific gene-sets with fewer than 9 genes. Seq2pathway loads this curated GO database because it has been widely used for gene-set enrichment analysis (GSEA)[17]. Seq2pathway also includes gene-sets derived from R object "org.Hs.egGO2EG_2.14.0" based on GO.db_2.14.0.

Table 3: Statistics about the case study used gene-set databases.

Database	Number of gene-sets	Number of gene symbols	Linkage for download	version
GO BP	9407	15034	GO.db_2.14.0; org.Hs.eg.db_2.14.0	09-15-14
GO MF	3529	15573	GO.db_2.14.0; org.Hs.eg.db_2.14.0	09-15-14
GO CC	1198	17204	GO.db_2.14.0; org.Hs.eg.db_2.14.0	09-15-14
curated GO	1454	8278	MSigDB_C5 v4.0	04-01-14
Disease	2135	7790	Human Disease Ontology	12-05-14
Reactome	1566	7359	Commons.7. Reactome.GSEA.hgnc	03-09-15

BP: biological process; MF: molecular function; CC: cellular component.

2.2 Highlights

Importantly, seq2pathway provides Bioconductor users a new tool to provide functional insight on NGS data systematically. We highlight the expected and novel results obtained only with seq2pathway (Table 4) followed by the R code to apply seq2pathway. In the main manuscript, we also compared the results of seq2pathway to that of Genomic Regions Enrichment of Annotations Tool (GREAT)[14] and ChIP-Enrich[22] for the ChIP-seq experiment.

Table 4: Common and fresh insights into NGS data using seq2pathway.

NGS	Ontology	Highlights
H3K27me3 enrichment	GO	Common: negative regulation of cell proliferation (GO:0008285)
	GO	New: RNA polymerase II distal enhancer sequence-specific DNA binding transcription factor activity (GO:0003705) [12]
Height-associated SNP	Disease<br>Reactome	Common: bone remodeling disease<br>Common: elastic fibre formation
	GO	New: In Utero Embryonic Development (GO:0001701) [25]<br>Common: Chondrocyte Differentiation (GO:0002062) [25][16]
High-risk AML	GO	Common: Immune Effector Process

The purpose of developing seq2pathway is to provide a novel and flexible Bioconductor tool for functional data mining. Users can apply, separately or jointly, a two-step algorithm consisting of "seq2gene" and "gene2pathway" components. In the case study using ChIP-seq data, we demonstrate use of functions `runseq2gene()` and `gene2pathway_test()` in series and a calling of the joint function `runseq2pathway()`.

2.3 ChIP-seq data analysis

2.3.1 Discover over-represented GO terms from H3K27me3 enriched peaks

`Seq2pathway` has the option to assign non-exon regions to a broader range of neighboring genes than the nearest one, facilitating the study of functional non-coding elements[27]. `Seq2pathway` then considers the contribution of sequence-level statistics to the gene-set enrichment scores.

2.3.2 Overcome the bias of gene length and gene-set size

Gene-set analyses are often prone to various sources of bias (e.g. gene length bias, gene set size bias, linkage disequilibrium patterns). We first show that, in this case study on ChIP-seq data, for gene-sets with five or more genes the calculation of empirical p-values is sufficient to address both potential issues (Figure 4). We observed similar patterns of empirical p-values repeating the simulation 1000 times or 100 times.

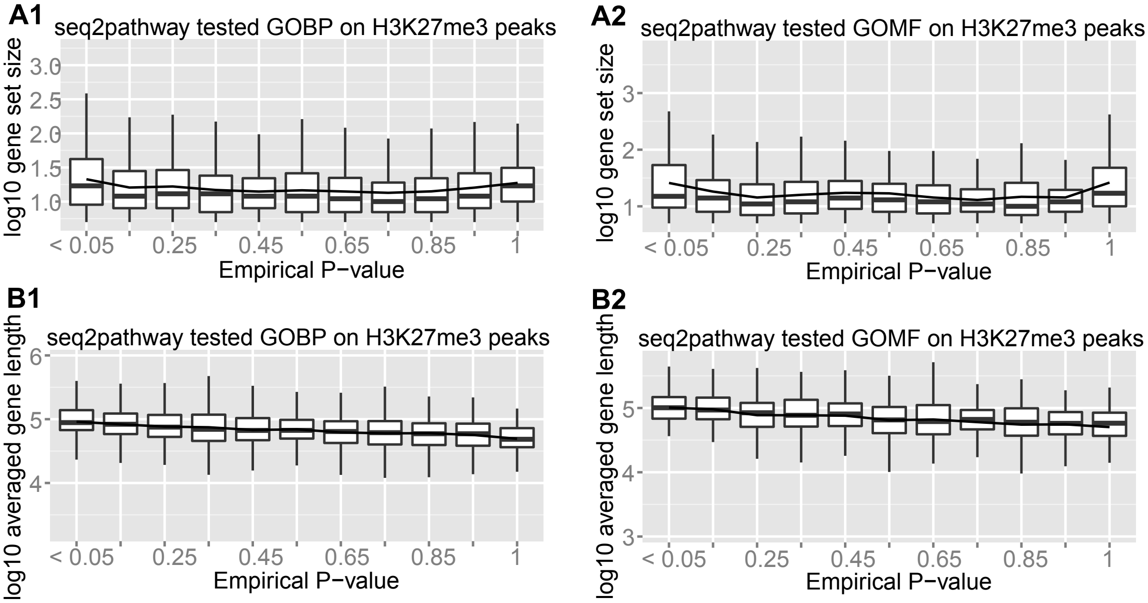

Figure 4: **Boxplots of gene-set sizes (panel A) and averaged lengths of genes within gene-sets (panel B) across different empirical p-value ranges**. We split the empirical p-values resulting from this case study into eleven bins. In each subpanel, the x-axis is the top value of each bin, and the y-axis is the log10 transformed gene-set size (top) or gene length (bottom). Each box shows the 25th and 75th percentiles of the data and the median. The "whiskers" extend to the most extreme data point within 1.5 times the interquartile range from the box. Additionally, a dotted line shows the average values across all boxes.

2.3.3 Method comparison

We looked into the H3K27me3 enriched loci in the GSM12878 cells downloaded from the ENCODE compendium. To make the seq2pathway analytic results comparable with the results of GREAT[14] and ChIP-Enrich[22], we set a 5k bp search radius.

Our results indicated that seq2pathway concentrates better with less identifications of significant GO terms than GREAT[14] or ChIP-Enrich[22] (Figure 5) and is the only method to reveal the significance of GO:0003705 (distal enhancer region for RNA polymerase II), a molecular function (MF) significantly enriched by bivalent genes marked with both H3K4me3 and H3K27me3 [12].

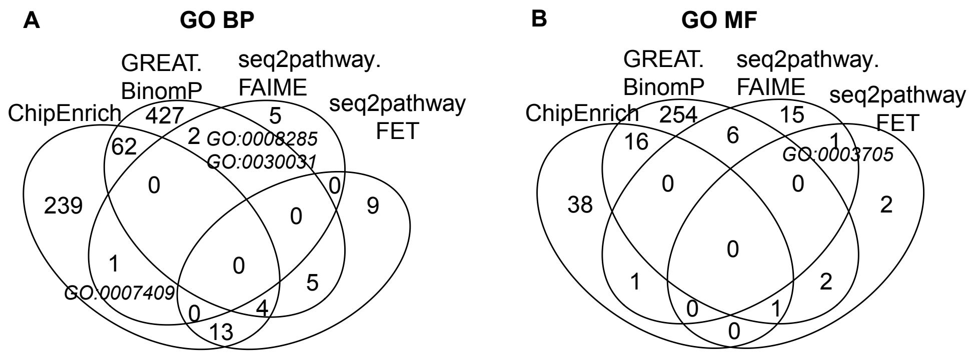

Figure 5: **Enriched biological process (BP) molecular function (MF) IDs identified by four methods.** We applied a search radius =5kb and limited the minimum gene-set size to five genes for all methods. Note that this simple comparison of GO ID overlapping is dependent on the background gene ontology (GO) used by different approaches (For details see Discussion).

2.3.4 R code

load ChIP-seq enriched peaks

```
> library("seq2pathway.data")
> library("seq2pathway")
> H3K27me3 <- read.table("result/mehod_comparison/data/GM12878_H3K27me3_Broad_Peaks.txt",
+               sep="\t",header=T)
> dim(H3K27me3)
[1] 41464     5
> H3K27me3[1:3,]
  peakID chrom    start      end signalvalue
1 Peak_1 chr22 16846641 16870328    3.326709
2 Peak_2 chr22 17309905 17319821    1.704024
3 Peak_3 chr22 17386956 17483002    1.964381
> dim(H3K27me3)
[1] 41464     5
```

Example 1: a joint run

In the seq2pathway package, runseq2pathway() is one of the key functions. The input file contains sequence-level measurements of genomic regions (including genomic coordinates and ChIP-seq enrichment scores or -log p-values), and the output is statistics on gene-set level.

```
# This step may be time-consuming and depends on the length of peaks
# and the parameter B used to estimate empirical P-value.
# Users can take the demo data in the seq2pathway package with a smaller B value
# for practice (see vignette).
>
> seq2pathway_5KB <- runseq2pathway(inputfile=H3K27me3,
+                     search_radius = 5000,
+                     genome = "hg19",
+                     FAIMETest=TRUE, B=1000,
+                     FisherTest=TRUE)
> save(seq2pathway_5KB,file="seq2pathway_5KB_fullresult.rData",compress=T)
```

```
>
> names(seq2pathway_5KB)
[1] "seq2gene_result"     "gene2pathway_result" "FS_test"
> names(seq2pathway_5KB$seq2gene_result)
[1] "seq2gene_FullResult"           "seq2gene_CodingGeneOnlyResult"
> names(seq2pathway_5KB$gene2pathway_result)
[1] "GO_BP" "GO_CC" "GO_MF"
```

Example 2: a separate run

In the seq2pathway package, `runseq2gene()` and `gene2pathway_test()` are the key functions. The input file of `runseq2gene()` is sequence-level measurements of genomic regions (including genomic coordinates and ChIP-seq enrichment scores or -log p-values), and the output is statistics on the gene level. The input file of `gene2pathway_test()` is gene-level measurements (runseq2gene outputs or RNA-seq measurements), and the output is statistics on the gene-set level.

```
# Alternatively, the user can call two steps separately with more flexibility.
>
>  seq2gene_5KB <- runseq2gene(inputfile=H3K27me3, search_radius=5000, genome="hg19")
> names(seq2gene_5KB)
[1] "seq2gene_FullResult"           "seq2gene_CodingGeneOnlyResult"
>
# This step takes a while for large data sets by calling function collapseRows() in the R package{WGCN
>
> dat_collapsed <- Peak_Gene_Collapse(input = peak_anno_score,collapsemethod = "MaxMean")
> dim(dat_collapsed)
[1] 15972     2
> dat_collapsed[1:3,]
         peakID signalvalue
A1BG  Peak_6163    2.692105
A1CF  Peak_3444    7.913647
A2M  Peak_31218    8.162426
> save(dat_collapsed, file="seq2gene_5KB_collapsed_03102015.rData",compress=T)
>
> dat_CP <- data.frame(dat_collapsed[, c(2:ncol(dat_collapsed))])
> rownames(dat_CP) <- rownames(dat_collapsed)
> colnames(dat_CP) <- colnames(dat_collapsed)[2:ncol(dat_collapsed)]

# This step takes a while for large data sets when EmpiricalTest=TRUE #
>
>  GO_BP_FAIME <- gene2pathway_test(dat = dat_CP, DataBase = GO_BP_list,
+                 method = "FAIME", genome="hg19",
+                 FisherTest=FALSE, EmpiricalTest=TRUE)
>  colnames(GO_MF_FAIME)[3] <- "signalvalue2pathscore_Pvalue"
>  save(GO_MF_FAIME,file="seq2gene_5KB_GO_MF_FAIME_03102015.rData",compress=T)
```

For method comparison, we ran the following R code to get the enriched GO terms using R package `chipenrich`[22] and used the online tool GREAT[14]. We expect seq2pathway to recapture established knowledge and provide novel insights into the ChIP-seq data.

```
> library(chipenrich)
> result_5KBfrom_tss <- chipenrich(H3K27me3bed, genome = "hg19",
+               genesets =c('GOBP','GOCC','GOMF'), locusdef = "5kb",
+               method = "chipenrich", fisher_alt = "two.sided", use_mappability = F,
+               qc_plots = T)
> dim(result_5KBfrom_tss$peaks)
[1] 6606
> length(unique(result_5KBfrom_tss$peaks$gene_symbol))
[1] 3944
> save(result_5KBfrom_tss,file="result_5KBfrom_tss.rData")
>
# The common identification between seq2pathway and GREAT tools.
>
> FAIME_BP_Empirical_FDR[which(rownames(FAIME_BP_Empirical_FDR) %in% GREAT_BP_BinomP$ID),][,c(1,3,4)]
                                                                                          Des
GO:0008285   Any process that stops, prevents or reduces the rate or extent of cell proliferation.
GO:0030031 Formation of a prolongation or process extending from a cell, e.g. a flagellum or axon.
           signalvalue2pathscore_Pvalue Intersect_Count
GO:0008285                            0             311
GO:0030031                            0               7
>
> x <- which(rownames(FAIME_MF_Empirical_P) %in% GREAT_MF_BinomP$ID)
> FAIME_MF_Empirical_P[x,][,c(1,3,4)]
[1] "Interacting selectively and non-covalently with double-stranded DNA."
[2] "Catalysis of the reactions: ATP + protein serine = ADP + protein serine phosphate,
and ATP + protein threonine = ADP + protein threonine phosphate."
[3] "Catalysis of the reaction: protein + ATP = protein phosphate + ADP. This reaction
is the phosphorylation of proteins. Mitogen-activated protein kinase; a family of protein
kinases that perform a crucial step in relaying signals from the plasma membrane to the
nucleus. They are activated by a wide range of proliferation- or differentiation-inducing signals;
activation is strong with agonists such as polypeptide growth factors and tumor-promoting phorbol
esters, but weak (in most cell backgrounds) by stress stimuli."
[4] "Interacting selectively and non-covalently with ATP, adenosine 5'-triphosphate, a
universally important coenzyme and enzyme regulator."
[5] "Catalysis of the transmembrane transfer of a calcium ion by a channel that opens
when a specific ligand has been bound by the channel complex or one of its constituent parts."
[6] "Catalysis of the reaction: MAP kinase serine/threonine/tyrosine phosphate + H2O =
MAP kinase serine/threonine/tyrosine + phosphate."
>
> x <- which(rownames(FAIME_BP_Empirical_FDR) %in% ChipEnrich_BP_FDR$Geneset.ID)
> FAIME_BP_Empirical_FDR[x,][,c(1,3,4)]

GO:0007409 Generation of a long process of a neuron, that carries efferent (outgoing) action
potentials from the cell body towards target cells.
           signalvalue2pathscore_Pvalue Intersect_Count
GO:0007409                            0              66
>

# Users also have a chance to obtain novel insight through application of approaches
# provided in the seq2pathway package.
>
>  FAIME_BP_Empirical_FDR$Des
[1] "The process whose specific outcome is the progression of the embryo in the uterus
over time, from formation of the zygote in the oviduct, to birth. An example of this process
```

```
is found in Mus musculus."
[2] "The last step in the formation of the neural tube, where the paired neural folds are
brought together and fuse at the dorsal midline."
[3] "Generation of a long process of a neuron, that carries efferent (outgoing) action
potentials from the cell body towards target cells."
[4] "Any process that stops, prevents or reduces the rate or extent of cell proliferation."
[5] "Formation of a prolongation or process extending from a cell, e.g. a flagellum or axon."
[6] "Any process that affects the structure and integrity of a protein by altering the
likelihood of its degradation or aggregation."
[7] "Any process that activates or increases the frequency, rate or extent of transcription
elongation, the extension of an RNA molecule after transcription initiation and promoter
clearance by the addition of ribonucleotides, catalyzed by RNA polymerase II."
[8] "Any cell cycle regulatory process that controls the commitment of a cell from G1 to
S phase of the mitotic cell cycle."
>
```

2.4 SNP data analysis

Great advances have been made in the field of genetic analysis over the last decades, while the availability of millions of single nucleotide polymorphism (SNP) data has increased. However, studies elucidating the genetic basis of complex disease were cherry-picking, and we lack systematic analytic tools for this process. In this case study, we show that by applying seq2pathway on SNP data, we can recapture the "bone development" related GO terms discovered by GREAT[14] and reveal new pathways (eg, elastic fibre formation, bone resorption disease).

2.4.1 Discover over-represented Disease Ontology terms from height-associated SNPs

Specifically, we searched "height" trait from NHGRI compendium on February 2015. We got 521 SNPs from 21 studies and innovatively apply the seq2pathway approach on functional analysis of 460 height-associated human SNPs with reported p-values.

We demonstrate here the use of the seq2pathay approach to discover over-represented pathways defined in the Reactome database, human disease ontologies, and other pre-defined functional gene-sets. Note that seq2pathway does not adjust for the effect of population size on meta-analysis.

2.4.2 Overcoming the bias of gene length, gene-size, and linkage disequilibrium

We again investigate the effect of gene length on empirical p-values using the FAIME method. We find that FAIME score adjusts gene-set bias but its empirical p-value is prone to favorite small gene-sets with 1 or 2 gene members (Figure 6 A1). This bias could be adjusted when applying this method on gene-sets with 3 or more genes (Figure 6 A2) and fully corrected for gene-set with five or more genes (Figures 6 A3, 4 A1). Additionally, gene-length bias doesn't affect the distribution of reported FAIME scores (Figure 6 D).

Additionally, As we collapse the significance of neighboring SNPs with preference to higher linkage disequilibrium (LD), our method should overcome the common bias of linkage disequilibrium in pathway analysis of SNPs. To test this hypothesis, we calculated the average European LD scores for SNP-SNP pairs that were mapped to any genes in a GO BP term. We used the online tool to derive LD scores (threshold at 0.2) from the 1000 Genomes Project Haploreg version 3[24]. All together, 433 SNPs were mapped to one or more GO BP genes. Although all averaged LD scores per GO term are higher than 0.6, the distribution of these LD scores has no significant bias in terms of gene-set enrichment (Figure 6 C). We observed the similar results for another ethnic population which is as expceted, as the human height trait-SNPs were not collected from ethnic specific GWAS.

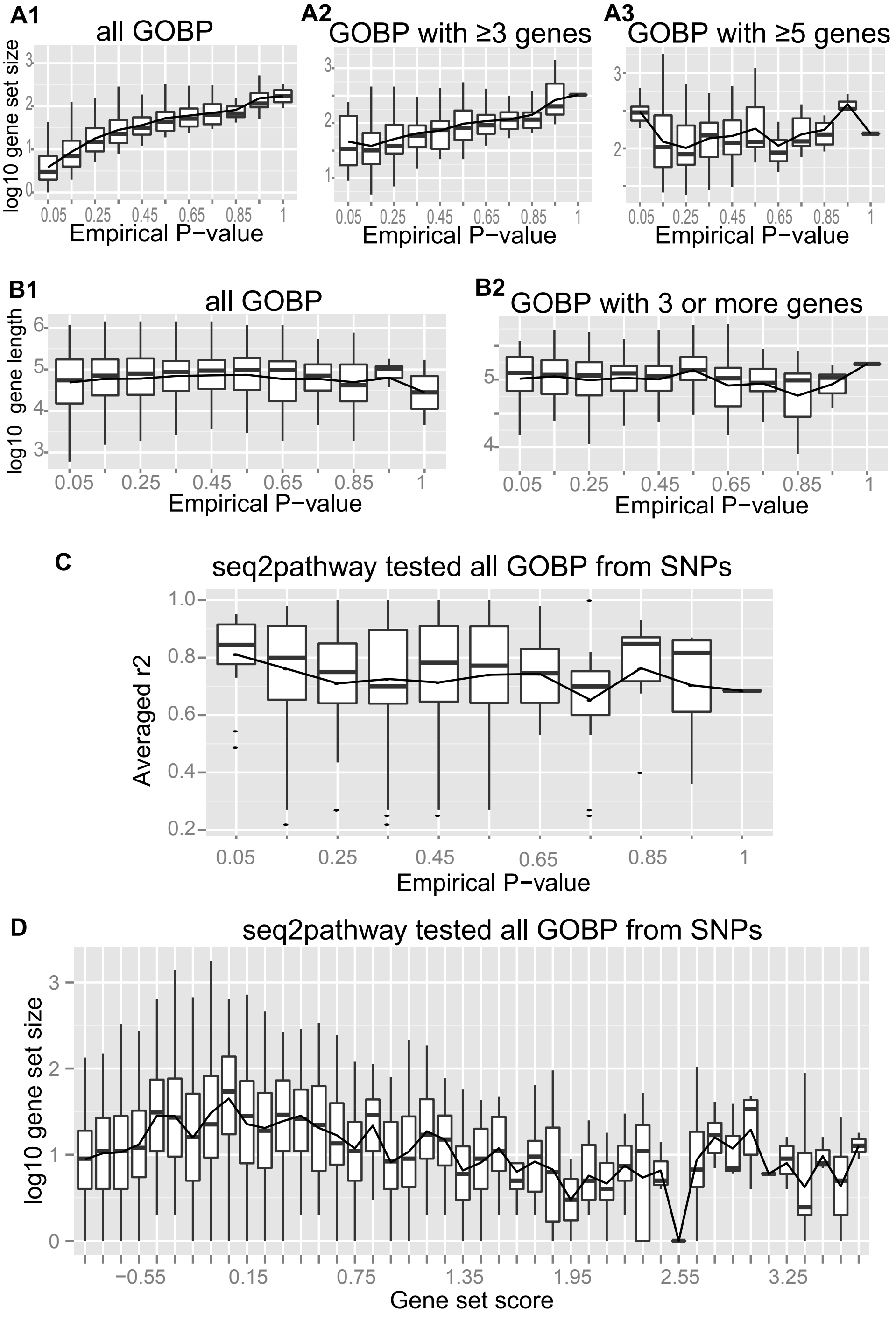

Figure 6: **Boxplots of gene-set sizes (panel A), the average lengths of genes within gene-sets (panel B), and average linkage disequilibrium of SNP pairs (panel C) across different empirical p-value ranges. Additionally, Panel D is the boxplot of gene-set sizes across different FAIME score ranges.** We split the empirical p-values into eleven bins and the FAIME scores into 44 bins. In each subpanel, the x-axis is the top value of each bin, and the y-axis is the log10 transformed gene-set size (A, D), gene length (B), or averaged LD scores for any pair of SNPs that were mapped to genes in the same GO BP (C).

2.4.3 Method comparison

For the method comparison, we ran the GREAT online tool on these 460 human height-associated SNP loci and compared the output with the seq2pathway results (using the default searching parameter, BinomFdrQ < 0.05, observed regions > 3, and region fold enrich > 2).

Again, we found seq2pathway is more sensitivity than GREAT for test of gene-set enrichment in terms of the number of identified GO terms (Figure **??**). Additionally, 14% of seq2pathway pathway-score identified GO terms recapture previously predicted height-associated biological processes, more than GREAT (0.6%) did in this case study.

Notably, four out of the six most top enriched diseases ontology terms (FET, count >3, $p < 0.01$) are related directly to bone disorder, including bone remodeling disease, arthropathy, osteoporosis, and bone resorption disease. Compared to the GREAT report on the same SNP list, the new insights into "bone resorption disease" and "elastic fibre formation" are caused by our new seq2gene mapping method[27] and the flexibility to analyze gene-set databases of interests. We found that seq2pathway applying both the hypergeometric test (theoretic $p < 0.05$) and the FAIME scores (empirical $p < 0.01$) recaptures the expected bone development associated biological processes and cell components, such as "Chondrocyte Differentiation" (GO:0002062)[16].

Interestingly, the valuable biological processes obtained only with seq2pathway (empirical $p < 0.05$) include "In Utero Embryonic Development" (GO:0001701) that was predicted by a comprehensive study on human height[25] (Figure 1 in the main manuscript).

2.4.4 R code

load ontology

```
> library("GSA")
> Recon <- GSA.read.gmt("Pathway Commons.7.Reactome.GSEA.hgnc.gmt")
> names(Recon)
[1] "genesets"              "geneset.names"        "geneset.descriptions"
> dim(Recon$genesets)
NULL
> length(Recon$genesets)
[1] 1566
> load("pathway_download/HumanDisease/DiseaseOntology.db.symbols.gmt.rData")
> class(DiseasOntology)
[1] "GSA.genesets"
> length(DiseasOntology[[1]])
[1] 2135
```

Example 3: analysis of SNP data

```
> bed <- read.table("Height_GWASSearch_4seq2pathway.txt",sep="\t")
> dim(bed)
[1] 460   5
> bed[1:3,]
        SNPs  chr     start       end Pvalue_mlog
1 rs10010325 chr4 105185196 105185197    10.39794
2 rs10037512 chr5  89058858  89058859    17.69897
3 rs10058074 chr5 132350453 132350454    11.39794

>  res.GO <- runseq2pathway(inputfile=bed,
+               search_radius=5000,        # be comparable to GREAT
+               genome="hg19", SNP= TRUE,
```

```
+               DataBase="GOterm",
+               FAIMETest=TRUE, min_Intersect_Count=1)   # be comparable to GREAT
>
>  res.Reactome <- runseq2pathway(inputfile=bed,
+               search_radius=5000,
+               genome="hg19", SNP= TRUE,
+               DataBase=Recon,
+               FAIMETest=TRUE, min_Intersect_Count=1)
>
>  res.Disease <- runseq2pathway(inputfile=bed,
+               search_radius=5000,
+               genome="hg19", SNP= TRUE,
+               DataBase=Disease,
+               FAIMETest=TRUE, min_Intersect_Count=1)
>  save(res.GO, file="seq2pathway_OR.output.rData",compress=T)
>
```

Highlighted results

```
# FET found bone development related GO terms.
>
> minCount = 3
> FS_BP_test <- res.GO$gene2pathway_result.FET$GO_BP
> FS_BP_test <- FS_BP_test[FS_BP_test$Intersect_Count>=minCount,]
> seq2pathway_BP_FET_P <- FS_BP_test[FS_BP_test$Fisher_Pvalue<=0.01,]
> seq2pathway_BP_FET_P[6,]
       GOID
6 GO:0001649
  Description
6 The process whereby a relatively unspecialized cell acquires the specialized features of
an osteoblast, a mesodermal or neural crest cell that gives rise to bone.
  Fisher_Pvalue Fisher_odds       FDR Intersect_Count GO_gene_inBackground GO_gene_raw_Count
6  0.0007785541     4.56661 0.1450358               8                   55                55
                              Intersect_gene
6 BMP2 BMP6 RUNX2 IGFBP5 MEF2C MSX2 NF1 WNT11
>
> seq2pathway_BP_FET_P[9,]
        GOID
11 GO:0030501
                                                                                     Description
11 Any process that activates or increases the frequency, rate or extent of bone mineralization.
   Fisher_Pvalue Fisher_odds       FDR Intersect_Count GO_gene_inBackground GO_gene_raw_Count
11  0.0007580774    6.424759 0.1450358               6                   31                31
                    Intersect_gene
11 BMP2 BMP6 BMPR2 FBN2 MEF2C CD276
>
# However, FAIME finds no significant GO term at the significance level of empirical p<0.01.
# But with a moderate significance (p<0.05), FAIME can find two valuable GO terms predicted by
# a comprehensive study on human height\cite{Wood14}.
>
> FAIME_BP <- res.GO$gene2pathway_result.FAIME$GO_BP
> FAIME_BP <- FAIME_BP[FAIME_BP$Intersect_Count>=minCount,]
> FAIME_BP_Empirical_P <- FAIME_BP[FAIME_BP[,3]<=0.01,]
> nrow(FAIME_BP_Empirical_P)
```

```
[1] 0
> FAIME_BP_Empirical_P <- FAIME_BP[FAIME_BP[,3]<=0.05,]
> nrow(FAIME_BP_Empirical_P)
[1] 14
> cbind(Term(rownames(FAIME_BP_Empirical_P)),FAIME_BP_Empirical_P[,c('Intersect_gene','Pvalue_mlog2pat
                       Term(rownames(FAIME_BP_Empirical_P))
GO:0000082             G1/S transition of mitotic cell cycle
GO:0001701                    in utero embryonic development
GO:0001837              epithelial to mesenchymal transition
GO:0002062                       chondrocyte differentiation
GO:0006397                                   mRNA processing
GO:0006979                     response to oxidative stress
GO:0008380                                      RNA splicing
GO:0010596 negative regulation of endothelial cell migration
GO:0010629           negative regulation of gene expression
GO:0019882              antigen processing and presentation
GO:0040007                                            growth
GO:0043116       negative regulation of vascular permeability
GO:0048712  negative regulation of astrocyte differentiation
GO:0050919                               negative chemotaxis
                                      Intersect_gene Pvalue_mlog2pathscore_Pvalue
GO:0000082                      CDK6 ID4 PSMD1 RPS27A                        0.032
GO:0001701 BMP2 GNA12 MAN2A1 MECOM MKL2 PKD1 SLIT2 TANC2                        0.013
GO:0001837                            BMP2 HMGA2 SOX9                        0.039
GO:0002062                           BMP2 HMGA2 MEF2C                        0.017
GO:0006397       CELF5 CPSF6 FIP1L1 SF3A2 SNRNP48 TSEN15                        0.020
GO:0006979                             MICB MPO MSRB3                        0.020
GO:0008380                      HNRNPA3 SF3A2 SNRNP48                        0.037
GO:0010596                       APOH BMP10 SLIT2 SP100                        0.049
GO:0010629                CITED2 ESR1 MEF2C SLIT2 SLIT3                        0.049
GO:0019882                            MICA MICB PROCR                        0.029
GO:0040007                             BMP2 BMP3 BMP6                        0.033
GO:0043116                    PDE3A PTPRJ RAMP2 SLIT2                        0.049
GO:0048712                       HMGA2 ID4 NF1 NTRK3                        0.037
GO:0050919                           NRP2 SLIT2 SLIT3                        0.032
> intersect(rownames(FAIME_BP_Empirical_P), Wood14$Re.annotated.gene.set.ID)
[1] "GO:0001701" "GO:0002062"
>
# The most top diseases ontology term (count > 5, P < 0.01) is bone remodeling disease.
>
> FS_Disease_test <- res.Disease$gene2pathway_result.FET
> FS_Disease_test <- FS_Disease_test[FS_Disease_test$Intersect_Count>=minCount,]
> seq2pathway_Disease_FET_P <- FS_Disease_test[FS_Disease_test$Fisher_Pvalue<=0.01,]
> int <- seq2pathway_Disease_FET_P[seq2pathway_Disease_FET_P$Intersect_Count>5,]
> int[order(int$Fisher_Pvalue, decreasing=F),][1:6,c(1:4,6:7)]
        GeneSet                              Description Fisher_Pvalue Fisher_odds
17 DOID:0080005                   bone remodeling disease   0.001045791    2.834866
11    DOID:1205 hypersensitivity reaction type I disease   0.001064183    2.965863
4      DOID:381                               arthropathy   0.003144825    3.050060
9     DOID:3213                      demyelinating disease   0.003744924    2.440134
18   DOID:11476                              osteoporosis   0.004301768    2.904946
19 DOID:0080011                   bone resorption disease   0.004637818    2.870777
   Intersect_Count MsigDB_gene_inBackground
17              14                      137
```

```
11              13                       122
4               10                        91
9               15                       168
18              10                        95
19              10                        96
>
# The most top Reactome pathway (count > 5, P < 0.01) is elastic fibre formation.
>
> FS_Reactome_test <- res.Reactome$gene2pathway_result.FET
> FS_Reactome_test <- FS_Reactome_test[FS_Reactome_test$Intersect_Count>=5,]
> seq2pathway_Reactome_FET_P <- FS_Reactome_test[FS_Reactome_test$Fisher_Pvalue<=0.01,]
> nrow(seq2pathway_Reactome_FET_P)
[1] 11
> seq2pathway_Reactome_FET_P[1,-2]
                                          GeneSet Fisher_Pvalue Fisher_odds       FDR
1                    9606: Elastic fibre formation  0.0010398645    5.056402 0.4622198
  Intersect_Count MsigDB_gene_inBackground MsigDB_gene_raw_Count
1               7                       41                    41
                          Intersect_gene
1 BMP10 LTBP1 LTBP2 LOXL4 FBN2 BMP2 EFEMP1
>
# Seq2pathway can output more details to facilitate the follow-up studies.
>
> colnames(int)
[1] "GeneSet"                  "Description"              "Fisher_Pvalue"
[4] "Fisher_odds"              "FDR"                      "Intersect_Count"
[7] "MsigDB_gene_inBackground" "MsigDB_gene_raw_Count"    "Intersect_gene"
>
# User can track the details of contributing SNPs and likely target genes for
# each GO term of interest.
>
> FAIME_BP_Empirical_P["GO:0006397",]

GO:0006397 Any process involved in the conversion of a primary mRNA transcript into one or more mature
           Pvalue_mlog2pathscore_Normalized Pvalue_mlog2pathscore_Pvalue
GO:0006397                         1.089599                         0.02
           Intersect_Count                         Intersect_gene
GO:0006397               6 CELF5 CPSF6 FIP1L1 SF3A2 SNRNP48 TSEN15
                                                       Intersect_element
GO:0006397 rs7507204 rs10748128 rs17690232 rs12982744 rs3812163 rs1046934
>
```

2.5 RNA-seq data analysis

One advantage of the seq2pathway tool is that the pathway-level scores for each sample (hereafter termed profile) are systematic in the view of genome and individualized in the view of phenotype. This profile is individualized because the FAIME (Functional Analysis of Individual Microarray (or RNA-seq) Expression) algorithm[26] calculates the cumulative effects of genes inside a gene-set with the effects of those outside. The analysis of gene-sets is therefore superior to single-gene analysis in regard to noise and dimension reduction, as well as its desired biological interpretability.

We have recently systematically applied gene2pathway to a transcriptomic meta-analysis of samples from different laboratories [28]. In this case study, we demonstrate a simple two-group comparison of profiles between high-risk (n=10) and intermediate-risk (n=20) AML patients with cytogenetic abnormalities.

2.5.1 AML samples

We downloaded the available gene level RNA-seq data for 179 adult cases of de novo acute myeloid leukemia (AML) from the TCGA (The cancer genome atlas) on April 2014. The clinical information of these patients was obtained from the authors[19]. This data set was truncated into a simulation dataset and wrapped in the `seq2pathway` package. Following is demo code to run `gene2pathway_test` function to calculate an individualized gene-set score for MSigDB curated GO[17].

2.5.2 Gene-set profile is independent of gene-set size

To verify the effects of gene length and genes-set size, we also performed the calculation on real data from 30 AML samples. We found no bias of gene-set size or gene length on the output gene-set scores calculated from RNA-seq data (Figure 7).

2.5.3 Discover risk-dependent GO terms from AML patients with cytogenetic abnormalities.

As a demo case study, we simply call limma package to compare high-risk (n=10) and intermediate-risk (n=20) AML patients with cytogenetic abnormality. Not surprisingly, the FAIME algorithm found 10 curated GO terms, including "ACTIVATION OF IMMUNE RESPONSE" and "HOMOPHILIC CELL ADHESION".

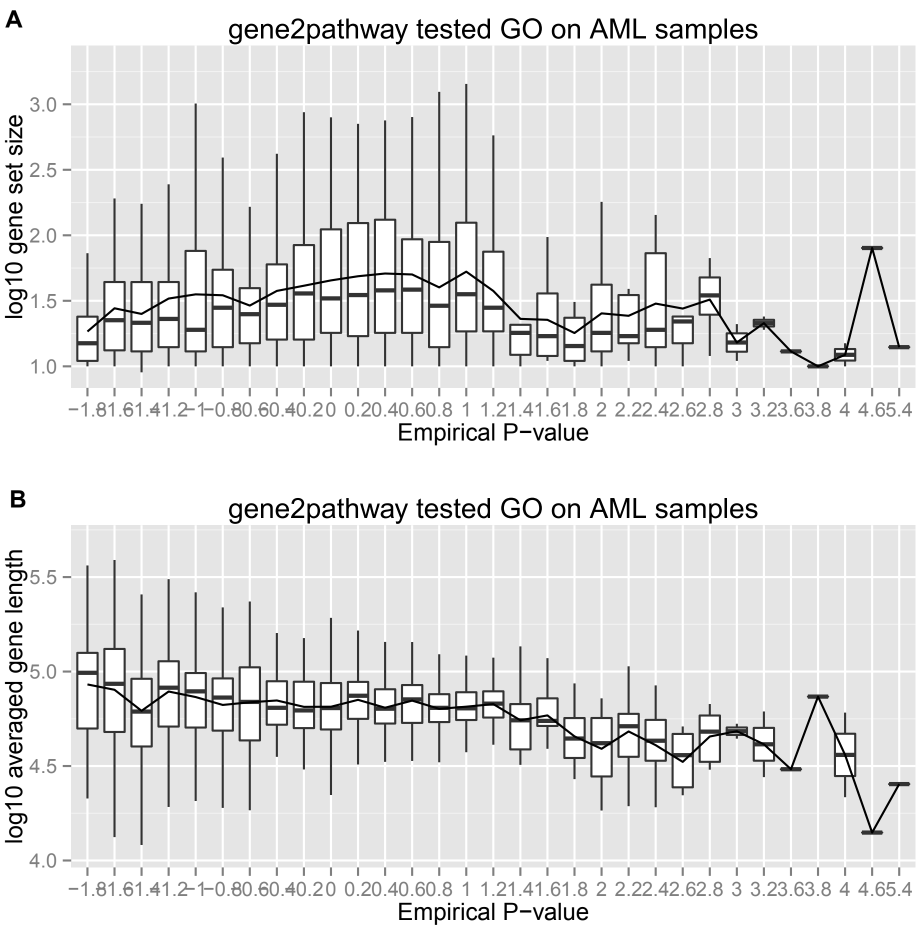

Figure 7: **Boxplots of gene-set sizes (Panel A) and average lengths of genes within gene-sets (Panel B) across different GO score ranges.** We split the gene2pathway output scores into eleven bins and the FAIME scores into 31 equal-sized bins. In each subpanel, the x-axis is the top value of each bin, and the y-axis is the log10 transformed gene-set size or gene length.

2.5.4 R code

RNA-seq is increasingly used for measuring gene expression levels. Normally, RNA-seq measures multiple samples from more than one sample-group. Based on expression on the gene-level, users can run the `gene2pathway_test` function and skip the `runseq2gene()` function.

Example 4: Pseudo-analysis of demo RNA-seq data

The inputs of the `gene2pathway_test` function running RNA-seq data will be a matrix of gene values and the output will be a matrix of pathway scores for multiple samples.

```
> data(dat_RNA)
> head(dat_RNA)
```

	TCGA_2841	TCGA_2840	TCGA_2843	TCGA_2842	TCGA_2845
A1BG	6.3606	10.2275	1.7113	1.7367	4.7184
A1BG-AS	8.7010	10.7700	2.5394	2.8203	7.8670
A1CF	0.0000	0.0000	0.0000	0.0000	0.0000
A2LD1	1.2489	1.3508	2.1397	1.9969	1.0495
A2M	0.2507	2.4767	3.3813	0.6906	1.7197
A2ML1	0.0710	0.0473	0.2541	0.0538	0.1098

```
> data(MsigDB_C5)
> dat_gene2path_RNA <- gene2pathway_test(dat=dat_RNA, DataBase=MsigDB_C5,
                          EmpiricalTest=F, method="FAIME", na.rm=T)
> head(dat_gene2path_RNA$gene2pathway_result.2)
```

Example 5: Analysis of real RNA-seq data

```
# Load the RNA-seq experiment data and sample information.
>
> load("RNAseq_data_preg2p.RData")
> dim(RNAseq_data_preg2p)
[1] 19990   179
> cli <- read.delim("SuppTable01.update.2013.05.13_simple.txt",sep="\t",header=T)
> dim(cli)
[1] 201  45
> cli <- cli[cli$RNAseq.data.=="Yes",]
> dim(cli)
[1] 179  45
>
# Select the patients with Poor Risk Cytogenetic Abnormality
>
> x <- cli$TCGA.Patient.ID[which(cli$Cytogenetic.Classification=="Poor Risk Cytogenetic Abnormality")]
> x <- paste("TCGA",x,sep="_")
> dat <- RNAseq_data_preg2p[,x]
> dim(dat)
[1] 19990    10
>
# Calculate GO scores for each samples
>
> dat_gene2path_RNA <- gene2pathway_test(dat=dat, DataBase=MsigDB_C5,
                          EmpiricalTest=F, method="FAIME", na.rm=T)
>  names(dat_gene2path_RNA)
[1] "gene2pathway_result.2"   "gene2pathway_result.FET"
> res <- dat_gene2path_RNA[[1]]
> dim(res)
[1] 1454   13
>
> res$GO_size <- sapply(MsigDB_C5[[1]], function(x) length(x))
> summary(res$GO_size)
   Min. 1st Qu.  Median    Mean 3rd Qu.    Max.
   9.00   15.00   27.50   92.43   71.75 2131.00
>
```

Example 6: Two-group comparison of gene-set scores

```
> library(limma)
> load(file="InterRiskCA.rData")
> dat <- dat_gene2path_RNA[[1]][,2:21]
> load(file="PoorRiskCA.rData")
> dat <- cbind( dat, dat_gene2path_RNA[[1]][,2:11])
> dim(dat)
[1] 1454   30
>
> f <- as.factor(c(rep("In",20),rep("Hi",10)))
> library(limma)
> design=cbind("In"=as.integer(f=="In"), "Hi"=as.integer(f=="Hi"))
> cont.matrix <- makeContrasts(Hi-In, levels=design)
> fit <- lmFit(dat, design)
> fit2 <- contrasts.fit(fit, cont.matrix)
> fit2 <- eBayes(fit2)
> output <- topTable(fit2, coef = 1.5)
> output[,1:4]
                                                  logFC     AveExpr         t      P.Value
SPERM_MOTILITY                                0.1524214 -1.14985772  3.911878 0.0004732316
REGULATION_OF_RESPONSE_TO_EXTERNAL_STIMULUS   0.2898786 -1.19845167  3.860267 0.0005454060
ACTIVATION_OF_IMMUNE_RESPONSE                -0.3515046 -0.02109307 -3.761998 0.0007137197
PROTEIN_AMINO_ACID_O_LINKED_GLYCOSYLATION     0.3060845  0.39423416  3.751432 0.0007345820
NF_KAPPAB_BINDING                             0.2691909  1.29714318  3.666778 0.0009245767
INSULIN_LIKE_GROWTH_FACTOR_RECEPTOR_BINDING  -0.4283254  0.30576260 -3.267470 0.0026768694
CELLULAR_MORPHOGENESIS_DURING_DIFFERENTIATION 0.1263386 -1.18689844  3.242681 0.0028555891
IMMUNE_EFFECTOR_PROCESS                      -0.1854022 -0.44508701 -3.220896 0.0030220385
HOMOPHILIC_CELL_ADHESION                      0.2269802 -0.74562459  3.120217 0.0039193253
CELLULAR_MACROMOLECULE_CATABOLIC_PROCESS     -0.1290443  1.25231258 -3.119784 0.0039236891
>
```

2.6 R environment session

> require(seq2pathway)

> sessionInfo();

```
R version 3.1.2 (2014-10-31)
Platform: x86_64-w64-mingw32/x64 (64-bit)

locale:
[1] LC_COLLATE=English_United States.1252
[2] LC_CTYPE=English_United States.1252
[3] LC_MONETARY=English_United States.1252
[4] LC_NUMERIC=C
[5] LC_TIME=English_United States.1252

attached base packages:
[1] stats     graphics  grDevices utils     datasets  methods   base

loaded via a namespace (and not attached):
[1] tools_3.1.2
```

3 Take home points

There are several take home points that users should pay attention to.

First, the background Gene Ontologies are different among different approaches (Figure 8). So when comparing methods, it is important to consider the content of GO terms rather than the size of overlap.

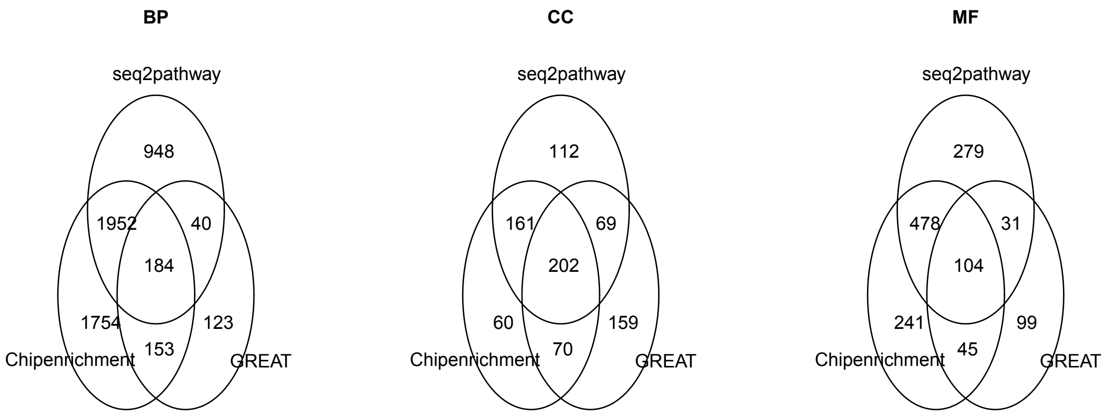

Figure 8: **Venn diagram of background GO IDs used in three gene-set enrichment analytic approaches.**

Second, we think that a customized filter on the "seq2gene" outputs for the study of likely cis-regulatory elementary element targets is critical when dealing with genomic region significance scores such as binding affinity estimates from ChIP-Seq.

Third, when applying gene2pathway analysis to selected gene list rather than the whole genome, one may user a smaller parameter α (by default is 5), allowing a moderate decay of weight from the most important genes to the least important genes in the list[28] (Figure 9).

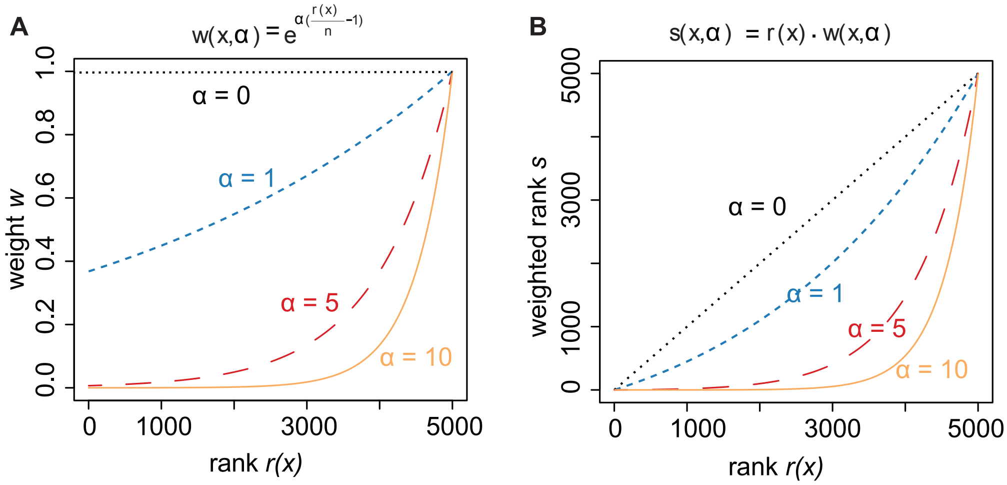

Figure 9: **The effects of the parameter α on the FAIME scores.** (A) Applying an increasingly larger α to the FAIME method. The weight (y-axis) is an exponential function of gene expression ranks (x-axis) adjusted by the parameter α. (B) Weight-dependent qualitative scores sharply increase with gene rank. The score (y-axis) is the product of gene expression ranks (x-axis) and the rank's weight adjusted by the parameter α. In each panel, the more highly expressed genes are ranked higher on the x-axis. The dashed line represents the score obtained with no weighting (i.e., ranking only)[28].

In summary,

- It is important to check and select the functional gene-set database before applying seq2pathway.
- Seq2pathway is suitable to test gene-sets with three or more genes.
- Seq2pathway overcomes common biases of SNP linkage disequilibrium, gene length, and gene-set size in pathway analysis of sequencing data.
- We suggest performing a customized filter on the "seq2gene" outputs for the study of likely cis-regulatory elementary element targets.
- In case novel functional element searching, we suggest a larger search radius ranging from 100 to 150 kbps, given that the average enhancer-promoter loop size is 120 kbps in mammalian genomes[3] and enhancers act in an orientation-independent manner[20].
- When applying gene2pathway analysis to selected gene list rather than the whole genome genes, one may user a smaller parameter alpha (rather than the by default value 5)[28].

4 References of the supplementary material

References

[1] G. K. Ardlie, L. Kruglyak, M. Seielstad, *Patterns of linkage disequilibrium in the human genome*, Nature Reviews Genetics **3** (2002), 299–309.

[2] Y. Benjamini, Y. Hochberg, *Controlling the False Discovery Rate: A Practical and Powerful Approach to Multiple Testing*, Journal of the Royal Statistical Society. Series B (Methodological) **57** (1995), 289–300.

[3] W. de Laat, D. Duboule, *Topology of mammalian developmental enhancers and their regulatory landscapes*, Nature **502** (2013), 499–506.

[4] T. Derrien, R. Johnson, G. Bussotti, A. Tanzer, S. Djebali, H. Tilgner, et al., *The GENCODE v7 catalog of human long noncoding RNAs: analysis of their gene structure, evolution, and expression*, Genome Res **22** (2012), 1775–1789.

[5] S. Durinck, Y. Moreau, A. Kasprzyk, S. Davis, B. De Moor, A. Brazma, et al., *BioMart and Bioconductor: a powerful link between biological databases and microarray data analysis*, Bioinformatics **21** (2005), 3439–3440.

[6] S. Durinck, P. T. Spellman, E. Birney, W. Huber, *Mapping identifiers for the integration of genomic datasets with the R/Bioconductor package biomaRt*, Nat Protoc **4** (2009), 1184–1191.

[7] N. D. Heintzman, B. Ren, *Finding distal regulatory elements in the human genome*, Current opinion in genetics & development **19** (2009), 541–549.

[8] Hoffmann A, Yang X, Burnicka-Turek O, Bosman J, Ren X, Hanson E, et al., *Foxf genes integrate Tbx5 and Hedgehog pathways in the second heart field for atrial septation*, PLoS Genetics **10(10)** (2014), e1004604.

[9] W. de Laat, D. Duboule, *Topology of mammalian developmental enhancers and their regulatory landscapes*, Nature **502** (2013), 499–506.

[10] E. S. Lander, L. M. Linton, B. Birren, C. Nusbaum, M. C. Zody, J. Baldwin, et al., *Initial sequencing and analysis of the human genome*, Nature **409** (2001), 860–921.

[11] A. Liberzon, A. Subramanian, R. Pinchback, H. Thorvaldsdottir, P. Tamayo, J. P. Mesirov, *Molecular signatures database (MSigDB) 3.0*, Bioinformatics **27** (2011), 1739–1740.

[12] Q. Li, S. Lian, Z. Dai, Q. Xiang, X. Dai, *BGDB: a database of bivalent genes*, Database: the journal of biological databases and curation **bat057** (2013).

[13] C. Lottaz, X. Yang, S. Scheid, R. Spang, *OrderedList-a bioconductor package for detecting similarity in ordered gene lists*, Bioinformatics **22** (2006), 2315–2316.

[14] C. McLean, D. Bristor, M. Hiller, S. Clarke, B. Schaar, C. Lowe, A. Wenger, G. Bejerano, *GREAT improves functional interpretation of cis-regulatory regions*, Nature biotechnology **28** (2010), 495–501.

[15] A. Perez-Rathke, H. Li, Y. Lussier, *Interpreting personal transcriptomes: personalized mechanismscale profiling of RNAseq data*, Pac Symp Biocomput. (2013) 159–170.

[16] T.H. Pers, J.M. Karjalainen, Y. Chan, H. Westra, A.R. Wood, et al., *Biological interpretation of genome-wide association studies using predicted gene functions*, Nature communications (2015) 6.

[17] A. Subramanian, P. Tamayo, V. K. Mootha, S. Mukherjee, B. L. Ebert, M. A. Gillette, et al., *Gene set enrichment analysis: a knowledge-based approach for interpreting genome-wide expression profiles*, Proc Natl Acad Sci USA **102** (2005), 15545–15550.

[18] van den Boogaard M, Smemo S, et al., *Initial sequencing and analysis of the human genome*, J Clin Invest. **124** (2014), 1844–1852.

[19] Cancer Genome Atlas Research Network, *Genomic and epigenomic landscapes of adult de novo acute myeloid leukemia*, N Engl J Med **368** (2013), 2059–2074.

[20] A. Visel, E. M. Rubin, L. A. Pennacchio, *Genomic views of distant-acting enhancers*, Nature **461** (2009), 199–205.

[21] J. D. Walton, D. R. Kattan, S. K. Thomas, B. A. Spengler, H. F. Guo, J. L. Biedler, et al., *Characteristics of stem cells from human neuroblastoma cell lines and in tumors*, Neoplasia **6** (2004), 838–845.

[22] R.P. Welch, C. Lee, R.A. Smith,S. Patil, T. Weymouth, P. Imbriano, L.J. Scott, M.A. Sartor, *ChIP-Enrich: Gene set enrichment testing for ChIP-seq data*, NAR. **42(13)** (2014),e105.

[23] X. Yang, K. Regan, Y. Huang, Q. Zhang, J. Li, T. Y. Seiwert, et al., *Single sample expression-anchored mechanisms predict survival in head and neck cancer*, PLoS Comput Biol **8** (2012), e1002350.

[24] L.D. Ward, M. Kellis, *HaploReg: a resource for exploring chromatin states, conservation, and regulatory motif alterations within sets of genetically linked variants*, Nat Genet. **40(Database issue)** (2012), D930–D934.

[25] A.R. Wood, T. Esko, J. Yang, S. Vedantam, T.H. Pers, S. Gustafsson, et al., *Defining the role of common variation in the genomic and biological architecture of adult human height*, Nucl. Acids Res. **46** (2011), 1173–1186.

[26] X. Yang, K. Regan, Y. Huang, Q. Zhang, J. Li, T. Y. Seiwert, et al., *Defining the role of common variation in the genomic and biological architecture of adult human height*, Nat Genet. **46(11)** (2014), 1173–86.

[27] X. Yang, B. Wang, J. M. Cunningham, *Dentification of epigenetic modifications that contribute to pathogenesis in therapy-related AML: Effective integration of genome-wide histone modification with transcriptional profiles*, BMC Medical Genomics **V8 S2:S6** (2015), TBC/ISB 2014.

[28] X. Yang, M. Li, B. Wang, W. Zhu, A. Desgardin, K. Onel, J. de Jong, J. Chen, L Chen, J. M. Cunningham, *Systematic computation with functional gene sets among leukemic and hematopoietic stem cells reveals a favorable prognostic signature for acute myeloid leukemia*, BMC Bioinformatics **16** (2015), 97.
